# Cost-Effectiveness Analysis of High-Risk Groups Tuberculosis Screening in Malaysia

**DOI:** 10.3389/fpubh.2021.699735

**Published:** 2021-07-12

**Authors:** Nor Zam Azihan Mohd Hassan, Asmah Razali, Mohd Ridzwan Shahari, Mohd Shaiful Jefri Mohd Nor Sham Kunusagaran, Juanita Halili, Nur Amalina Zaimi, Mohd Shahri Bahari, Farhana Aminuddin

**Affiliations:** ^1^Institute for Health Systems Research, Ministry of Health Malaysia, Shah Alam, Malaysia; ^2^Disease Control Division, Ministry of Health Malaysia, Wilayah Persekutuan Putrajaya, Malaysia; ^3^Medical Development Division, Ministry of Health Malaysia, Wilayah Persekutuan Putrajaya, Malaysia

**Keywords:** cost-effectiveness, tuberculosis, high-risk group, TB screening, TB program

## Abstract

Screening of high-risk groups for Tuberculosis (TB) is considered as the cornerstone for TB elimination but the measure of cost-effectiveness is also crucial in deciding the strategy for TB screening. This study aims to measure the cost-effectiveness of TB screening between the various high-risk groups in Malaysia. A decision tree model was developed to assess the cost-effectiveness of TB screening among the high-risk groups from a provider perspective using secondary data from the year 2016 to 2018. The results are presented in terms of an Incremental Cost-Effectiveness Ratio (ICER), expressed as cost per TB case detected. Deterministic and Probabilistic Sensitivity Analysis was also performed to measure the robustness of the model. TB screening among Person Living with Human Immunodeficiency Virus (PL HIV) was the most cost-effective strategy, with MYR 2,597.00 per TB case detected. This was followed by elderly, prisoners and smokers with MYR 2,868.62, MYR 3,065.24, and MYR 4,327.76 per one TB case detected, respectively. There was an incremental cost of MYR 2.49 per screening, and 3.4 TB case detection per 1,000 screening for TB screening among PL HIV in relation to TB screening among prisoners. The probability of symptomatic cases diagnosed as TB was the key driver for increasing cost-effectiveness efficacy among PL HIV. Results of the study suggest prioritization of high-risk group TB screening program by focusing on the most cost-effective strategy such as screening among PL HIV, prisoners and elderly, which has a lower cost per TB case detected.

## Introduction

Tuberculosis (TB) is a leading cause of morbidity and mortality, thus remains a key public health priority. It continues to kill more than a million people annually, despite the availability of effective medication with high cure rates since the 1960s (1). About two-thirds of global TB cases are in Western Pacific region, of which Malaysia is part of. Malaysia is classified as an intermediate TB burden country with a notification rate of <100 cases per 100,000 population (1).

Detection of active TB can either be done through mass screening or targeted screening, wherein, it focuses on selected high-risk groups (2). Based on published reports, various groups were identified as having a higher risk for TB and given priority in the TB screening program (3). Compared to the general population, the incidence of TB is higher among Person Living with Human Immunodeficiency Virus (PL HIV), alcoholics, drug abusers, etc. (3, 4). The prevalence of TB among high-risk groups was 0.5% (3).

Studies have found that the costs of TB diagnosis can range from as low as US Dollar (USD) 0.50 to as high as USD 175.00 depending on the diagnosis approach taken (5). While sputum smear was the lowest cost, an active case finding would result in a much higher cost. In addition, the cost of hospitalization in countries with low and medium TB burdens such as Malaysia is much higher compared to countries with high TB burdens (6). Hence, lots of resources are required to manage TB cases in those countries. The presence of HIV co-infection also would result in much higher costs compared to TB infection without co-infection (6).

Malaysia has initiated high-risk group screening since 2015 in line with World Health Organization (WHO) End TB Strategy recommendation. However, screening of TB contact has been implemented much earlier in Malaysia, since 2003 (7). The goal of TB control in Malaysia is to ensure universal access to diagnosis and treatment of TB and prevent drug resistance TB to reduce the TB disease burden in the country (7). One of the strategies is to enhance the case detection rate of TB by increasing the number of TB notifications to 100 per 100,000 populations and symptomatic TB screening to 2,000 per 100,000 population by 2020 (7).

For years, the Ministry of Health (MOH), Malaysia has been focusing on TB screening among those high-risk of developing TB. This includes close contacts to TB cases (both household and none-household contacts), immunocompromised patients such as those suffering from Diabetes Mellitus (DM), Rheumatoid Arthritis and PL HIV, substance abusers and cigarette smokers, living in overcrowded conditions such as incarceration and institutionalization (whether in Cure and Care Rehabilitation Centers (CCRC), residents of Elderly Nursing home, prisoners, etc.) and an elderly (8). Different countries might have a different approach to high-risk groups TB screening depending on the TB situation of that particular country. For example, some countries may include immigrants as part of high-risk groups screening, while others might opt for Xpert MTB/RIF assay for diagnosing TB in addition to the conventional chest X-ray (CXR) and sputum smear (9, 10).

CXR has been the main screening tool in Malaysia for diagnosing TB among the asymptomatic high-risk population (8). However, it is known to give an unreliable result when used for diagnosing TB among the asymptomatic (11). Whereas, for the symptomatic, both CXR and sputum smear remains the mainstay of TB screening tool (8). Based on a study done in Malaysia on CXR screening among the asymptomatic, HIV was found to have the highest yield (25%), followed by smokers (20.7%), End Stage Renal Failure (ESRF) (20%), individual with substance abuse (13.3%), diabetic patients (10.6%), institutionalized individual (7.2%), and close contacts of TB cases (4.4%) (12).

Despite that, for an effective TB screening program, prioritization of key interventions and target groups are necessary (13). Unsystematic and poorly targeted screening may not lead to the desired outcomes. In the contrary, it can be very expensive, and gives minute impact in TB case detection (13, 14). Hence, screening for active tuberculosis should target those with higher risk, while taking into account the measures of effectiveness (13).

For the past few years, MOH Malaysia has allocated a substantial amount of resources for TB screening among high-risk groups. However, till now, there has not been any economic analysis done for this program. Hence, this study aims to assess the cost-effectiveness of TB screening across high-risk groups, which would provide information about the cost-effectiveness of each screening strategy, at the same time providing information on the lowest cost and most effective TB screening strategy for optimal use of resources from the perspective of health care provider, namely, the Ministry of Health, Malaysia.

## Materials and Methods

A decision tree model was developed to estimate the relative cost-effectiveness measure of TB screening between different high-risk groups. Subsequently, costing and probability data were calculated and introduced into the model. The effectiveness parameters used were the probability of each screening strategy manage to detect one TB case. These take into the form of probability for the symptomatic or the asymptomatic screening for each high-risk group results in TB case detection. Data were obtained from various sources as shown in Table 1. The costs per screening, cost per TB case detected and incremental cost-effectiveness ratio (ICER) for each high-risk group were presented as the outcome of this study. The cost per screening for particular high-risk groups is the average cost to screen one individual from that particular high-risk group, taking into account both symptomatic and asymptomatic individuals. Cost per TB case detected refers to the cost to detect one TB case for that particular high-risk group. It derived from dividing the cost per screening by the number of TB cases detected per screening. Whereas, the ICER refers to the additional cost requires to increase one additional TB case detection when comparing one high-risk group to other high-risk groups. To assess the robustness of the model, sensitivity analysis was also conducted. All costs were valued in 2018 and presented in Malaysia Ringgit (MYR), wherein MYR 1.00 ~ USD 0.25 (15). The time horizon for this study is 1 year. Willingness-to-pay (WTP) was capped at MYR 120,000 as of 3 times GDP per capita as suggested by WHO (16, 17). In 2018, Malaysia GDP per capita was valued around MYR 40,000 (~USD 9,660) (18).

**Table 1:** Source of data.

**Data**	**Type of data**	**Source of data**
Capital cost	Secondary	Disease control section, MOH
Personnel cost	Secondary	Disease control section, MOH
Consumables cost	Secondary	Disease control section, MOH
TB screening for high-risk group	Secondary	TBIS 204S for year 2016 to 2018 from Sabah and Sarawak State Health Departments

*TBIS, Tuberculosis Information System; MOH, Ministry of Health*.

### Decision Tree Model

**Figure 1:**
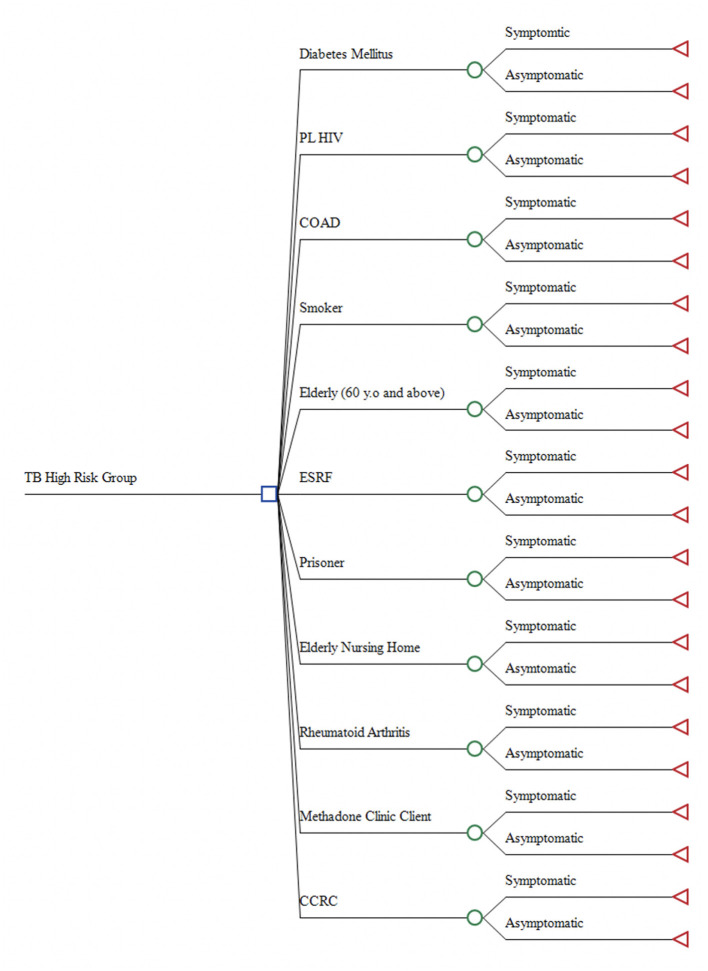
Decision Tree Model.

A Decision Tree Model was developed using TreeAge Pro version 2019 by TreeAge Software, Inc. (Figure 1). The model was constructed in concordance with the high-risk groups TB screening guideline from MOH Malaysia (8). A total of 11 mutually exclusive high-risk groups were included in the decision model, which were: (1) CCRC inmates; (2) Elderly Nursing Home residents; (3) ESRF patients; (4) Prisoners; (5) DM patients; (6) Methadone Clinic clients; (7) Rheumatoid Arthritis patient; (8) PL HIV; (9) Chronic Obstructive Airway Disease (COAD) patients; (10) Smokers, and; (11) Elderly (60 years and above). In this model, each high-risk group was branched out as symptomatic or asymptomatic. The symptomatic are those presented with typical TB symptoms such as productive cough, haemoptysis and chest pain, while asymptomatic are those presented without any TB symptoms (19).

Analysis of the decision tree model was executed based on few assumptions. Firstly, all screening procedures were assumed to be standardized, wherein, no significant variation in terms of the number of personnel, machinery, consumables used and times consumed. Thus, no difference in cost incurred despite different screening done in different settings. Secondly, the TB screening program is strictly following the guideline from MOH, in which the asymptomatic would only be screened through CXR, while the symptomatic is screened using both CXR and sputum smear or Sputum Acid Fast Bacilli (SAFB) test. This model also did not capture people who were false negative. Those negative and then developed symptoms will go through another TB screening procedure.

### Estimation for Probabilities of TB Case Detection

Secondary data on high-risk group TB screening was used to estimate probabilities of TB cases detected per screening of each high-risk group. This data was based on 3 years of Sabah and Sarawak State Health Department data on TB screening among high-risk groups, from 2016 to 2018 recorded in Tuberculosis Information System (TBIS) 204S for each State Health Departments. Cases of pending investigation results, referral to a specialist for TB diagnosis, TB diagnosis by other modalities than CXR and SAFB, and contact screening were excluded from this study. There was a total of 65,400 cases included for estimating the probabilities parameters. Probabilities parameters measured are shown in Table 2 consisted of the probabilities for the individual in each high-risk group having TB symptoms (i.e., being symptomatic). The probability values for the symptomatic high-risk group were calculated by dividing the number of each high-risk group presented with TB symptoms by the total number of TB screenings done for the respective high-risk group. Whereas, the probability values for asymptomatic is equal to one minus the probability value for the respective symptomatic high-risk group.

**Table 2:** Clinical input data for probabilities.

**Probability parameters**	**Probability value**	**Range^¶^**	**Distributions^§^**	**Alpha**	**Beta**
Symptomatic COAD patients	0.2132	0.1599–0.2665	Beta	12.38	45.67
Symptomatic CCRC inmates	0.0341	0.0256–0.0426	Beta	15.51	439.37
Symptomatic diabetes mellitus patients	0.0749	0.0562–0.0936	Beta	14.77	182.38
Symptomatic ESRF (haemodialysis)	0.0449	0.0337–0.0561	Beta	15.31	325.56
Symptomatic smokers	0.2198	0.1649–0.2748	Beta	12.24	43.45
Symptomatic PL HIV	0.2086	0.1565–0.2608	Beta	12.43	47.16
Symptomatic methadone clinic clients	0.0833	0.0625–0.1041	Beta	14.62	160.88
Symptomatic prisoners	0.0567	0.0425–0.0709	Beta	14.98	249.27
Symptomatic elderly nursing home residents	0.0416	0.0312–0.0520	Beta	15.29	352.32
Symptomatic rheumatoid arthritis patients	0.1875	0.1406–0.2344	Beta	12.80	55.46
Symptomatic Elderly (60 years and above)	0.2796	0.2097–0.3495	Beta	11.25	28.98

*COAD, Constrictive Obstructive Airway Disease; CCRC, Cure and Care Rehabilitation Center; ESRF, End Stage Renal Failure; PL HIV, Person Living with Human Immunodeficiency Virus; TB, Tuberculosis; na, not available*.

¶ *The probability parameter values are varied by ±25%*.

§ *Selection of distributions for each parameter are believed to be the best practice. Beta distribution is best used for probability value due to its properties, which ranges from 0 to 1*.

*The probability of asymptomatic equals to one minus the probability of symptomatic cases*.

### Estimation for Effectiveness Parameters

These effectiveness parameters used in this study were the number of TB cases detected per 1,000 screening for symptomatic and asymptomatic cases as shown in Table 3. The effectiveness parameters for the symptomatic were calculated by dividing the number of each high-risk group having presented with TB symptoms diagnosed as positive TB by the total number of the respective high-risk group having presented with TB symptoms (symptomatic). The results were then multiplied by 1,000 to get the value of TB cases detected per 1,000 screenings. A similar approach was also applied for the effectiveness parameters of the asymptomatic high-risk groups. Estimation of the effectiveness parameters for each high-risk group was calculated using the source of data from 2016 to 2018 Sabah and Sarawak State Health Department data on TB screening among high-risk groups recorded in TBIS 204S. From the total of cases screened, only 7,075 were symptomatic. A total of 288 from these symptomatic cases were positive TB. Whereas, only 177 of the asymptomatic were positive TB.

**Table 3:** Clinical input data for effectiveness.

**Effectiveness parameters**	**Value (TB case detected per 1,000 screening)**	**Range^¶^**	**Distributions^§^**	**Alpha**	**Beta**
**COAD patients**					
Symptomatic	20.4	15.3–25.5	Beta	15.65	751.66
Asymptomatic	1.8	1.4–2.3	Beta	12.93	7173.11
**CCRC inmates**					
Symptomatic	0.0	na	na	na	na
Asymptomatic	3.3	2.5–4.1	Beta	16.96	5121.28
**Diabetes mellitus patients**					
Symptomatic	23.4	17.6–29.3	Beta	15.34	640.15
Asymptomatic	1.5	1.1–1.9	Beta	14.04	9345.90
**ESRF (haemodialysis)**					
Symptomatic	75.3	56.5–94.1	Beta	14.76	181.2470
Asymptomatic	1.0	0.8–1.3	Beta	11.10	11087.90
**Smokers**					
Symptomatic	37.6	28.2–47.0	Beta	15.36	393.17
Asymptomatic	2.4	1.8–3.0	Beta	15.96	6633.71
**PL HIV**					
Symptomatic	57.5	43.1–71.9	Beta	15.18	248.84
Asymptomatic	6.1	4.6–7.6	Beta	16.43	2677.14
**Methadone clinic clients**					
Symptomatic	0.0	na	na	na	na
Asymptomatic	0.0	na	na	na	na
**Prisoners**					
Symptomatic	97.3	73.0–121.6	Beta	14.38	133.37
Asymptomatic	8.4	6.3–10.5	Beta	15.86	1871.90
**Elderly nursing home residents**					
Symptomatic	0.0	na	na	na	na
Asymptomatic	0.0	na	na	na	na
**Rheumatoid arthritis patients**					
Symptomatic	0.0	na	na	na	na
Asymptomatic	0.0	na	na	na	na
**Elderly (60 years and above)**					
Symptomatic	46.9	35.2–58.6	Beta	15.27	310.27
Asymptomatic	3.5	2.6–4.4	Beta	15.07	4289.80

¶ *The effectiveness parameter values are varied by ±25%*.

§ *Selection of distributions for each parameter are believed to be the best practice. Beta distribution is best used for effectiveness since this value also represents probability*.

### Estimation of Costs

This study only includes direct costs from the perspectives of MOH. This consists of capital, personnel and consumables costs (Table 1). The costs were calculated using a mix of step-down and Activity Based Costing (ABC) methods. Capital costs comprise medical equipment and yearly maintenance costs for both CXR and SAFB. Whereas, personnel costs include both staff's salaries and allowance per year based on the payslip and claim forms received from the administrative department. This was apportioned according to the duration it took to complete one whole procedure based on expert panels. Finally, the consumables costs consist of all materials used as part of the procedures.

In measuring the cost for conducting one symptomatic screening and asymptomatic screening, the cost for running one CXR and SAFB procedure was estimated. Based on the guidelines from MOH, the cost for one symptomatic screening is equal to the cost of one CXR and SAFB, while the cost for asymptomatic screening only consists of a cost for one CXR procedure (Table 4).

**Table 4:** Cost input data.

**Cost parameters**	**Unit cost (MYR)**	**Range (MYR)^¶^**	**Distributions^§^**
**Chest X-ray**			
Capital	5.12		
Personnel	31.56		
Consumables	3.59		
Total Cost for Chest X-Ray	40.27		
**Sputum AFB**			
Capital	3.18		
Personnel	5.07		
Consumables	8.13		
Total Cost for SAFB	16.38		
**Asymptomatic screening**			
Cost for chest X-ray	40.27	30.20–50.34	Gamma
Total	40.27		
**Symptomatic screening**			
Cost for chest X-ray	40.27	30.20–50.34	Gamma
Cost for SAFB	16.38	12.29–20.48	Gamma
Total	56.65		

*SAFB, Sputum for Acid Fast Bacilli*.

¶ *The cost parameters values are varied by ±25%*.

§ *All cost parameters are assigned with gamma distributions, which is the best practice. Gamma distribution is considered with parameters that have skewed distribution. It confined only to positive values and thus, is used in representing uncertainty for cost parameters*.

### Sensitivity Analysis

#### Deterministic Sensitivity Analysis

One-way sensitivity analysis was performed to assess the model's robustness toward change in parameters. Parameter values were changed with the corresponding minimum and maximum values, based on the range listed in Tables 2–4. The effect of each parameter change toward the ICER value was measured. Hence, the Tornado diagram is useful in identifying the key drivers for ICER values by demonstrating the changes in economic conclusions based on the variation of values of the selected parameters. Parameters that affect the ICER value the most are considered as the key driver for the ICER value. The result is demonstrated in the form of a Tornado Diagram as shown in Figure 4.

#### Probabilistic Sensitivity Analysis

Bayesian methods such as PSA are often used to measure the uncertainty effect of model parameters (20, 21). In this study, PSA was performed by assigning the model parameters with appropriate distributions model. The probabilities and costs parameters were allowed to varied and the effect of uncertainties was assessed by running a large number of simulations. PSA results are graphically demonstrated in the cost-effectiveness plane scatter diagram and Cost-Effectiveness Acceptability Curve (CEAC).

### Ethics Approval

The study was conducted according to the guidelines of the Declaration of Helsinki, and did not include any identifiable human data. The study had obtained approval from the Institutional Review Board (or Ethics Committee) of Ministry of Health Malaysia (NMRR-19-3443-51729).

## Results

**Figure 2:**
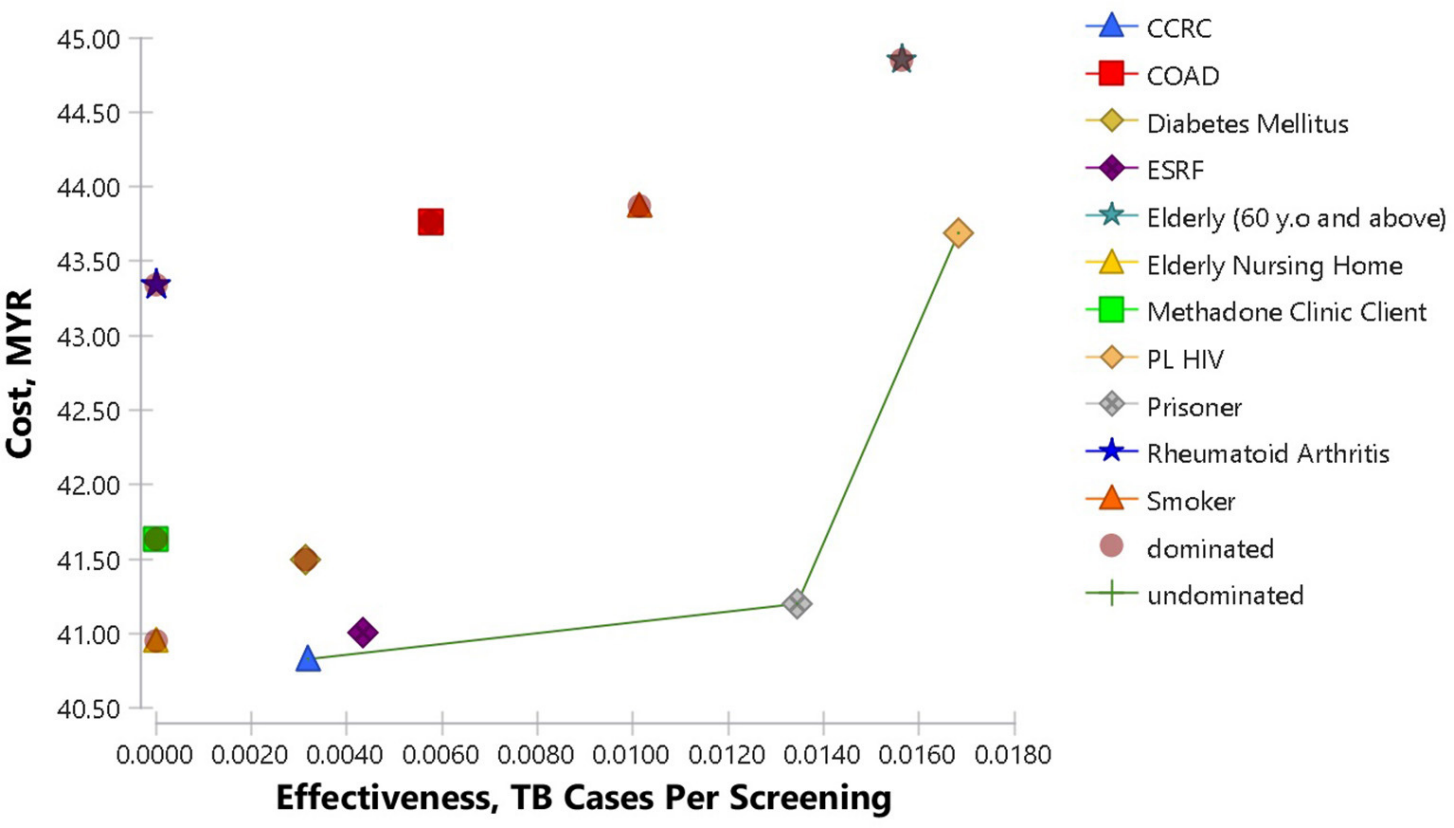
Cost-effectiveness Plane of TB screening among high-risk groups. The frontier is made up of CCRC, prisoners and PL HIV. The cost-effectiveness plane visualizes each strategy effectiveness and cost in relation to the others.

Results of cost-effectiveness analysis as shown in Table 5, consist of cost per TB screening, cost per TB case detected and the ICER. Figure 2 shows the cost-effectiveness plane of the analysis. TB screening among CCRC inmates had the lowest cost per screening with MYR 40.83, while TB screening among the elderly was the highest with MYR 44.85 per screening. The results also showed that TB screening among PL HIV is the most cost-effective strategy with MYR 2,597.00 per TB case detected. This was followed by a screening of TB among elderly, prisoners and smokers with MYR 2,868.62, MYR 3,065.24, and MYR 4,327.76 per TB case detected, respectively.

**Table 5:** Results of cost-effectiveness analysis of different high-risk group TB screening.

**Strategy**	**Cost per screening (MYR)**	**TB cases detected (per 1,000 screening)**	**Cost per TB case detected (MYR)**	**ICER**	
CCRC inmates	40.83	3.2	12,809.08	-	Dominant^*^
Elderly nursing home residents	40.95	0.0	na	−38.54	Dominated^**^
ESRF	41.01	4.3	9,456.83	154.02	Ext. Dominated^***^
Prisoner	41.20	13.4	3,065.24	21.23	Dominant^*^
Diabetes mellitus	41.50	3.1	13,214.26	−28.94	Dominated^**^
Methadone clinic client	41.63	0.0	na	−32.42	Dominated^**^
Rheumatoid arthritis	43.34	0.0	na	−159.41	Dominated^**^
PL HIV	43.69	16.8	2,597.00	735.82	Dominant^*^
COAD	43.76	5.8	7,590.33	−6.81	Dominated^**^
Smoker	43.87	10.1	4,327.76	−27.44	Dominated^**^
Elderly (60 years and above)	44.85	15.6	2,868.62	−979.43	Dominated^**^

*ICER, incremental cost-effectiveness ratio*.

* *Better outcomes, lower costs*.

** *Worse outcomes, higher costs*.

*** *Better outcomes, lower costs but the subsequent strategy has a positive ICER*.

As the initial reference point, TB screening among CCRC inmates would cost MYR 40.83 per screening, resulted in 3.2 TB case detection per 1,000 screening and costs MYR 12,809.08 per one TB case detected. TB screening among Elderly Nursing home residents revealed an increment of MYR 0.12 cost per screening and less 3.2 TB cases detected per 1,000 screening. This was a case of dominated strategy, providing worse outcomes but at a much higher cost. TB screening among ESRF patients on the other hand, resulted in extended dominated. Both the costs per screening and also TB cases detection increased by for MYR 0.18 and 1.1 per 1,000 screening, respectively. The subsequent strategy, which is TB screening among prisoners was a dominant case, with much better outcomes, even though the costs are slightly higher than screening the ESRF patients, resulting in positive ICER. Screening TB among prisoners increased the costs as much as MYR 0.19 but resulted in an increment of 9.1 TB case detection per 1,000 screening. The next dominant strategy was TB screening among PL HIV. This strategy resulted in increment costs of MYR 2.49 per screening, and 3.4 TB case detection per 1,000 screening in relation to TB screening among prisoners. The other strategies resulted in dominated cases with worse outcomes but at higher costs.

### Sensitivity Analysis

**Figure 3:**
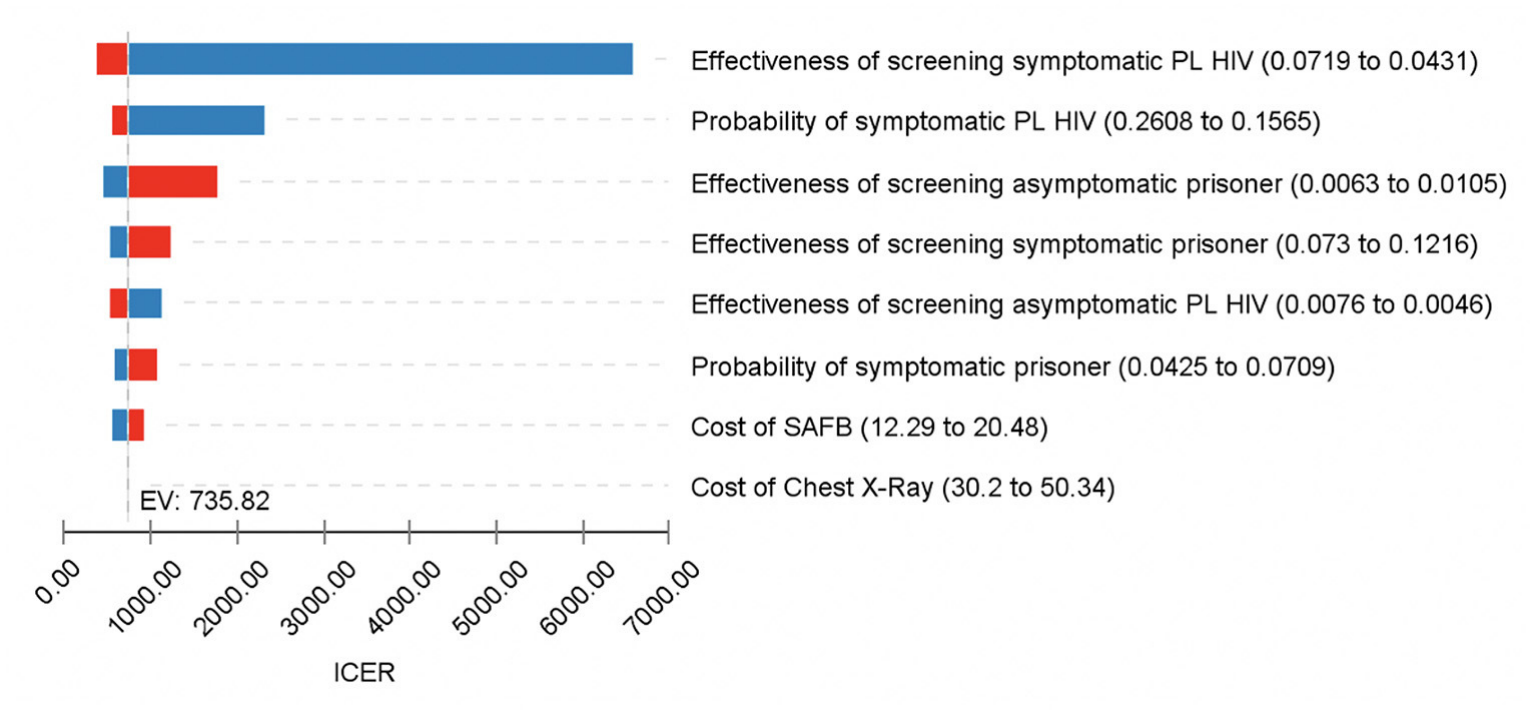
One-Way Sensitivity Analysis for TB screening among PL HIV vs. prisoners; Tornado diagram of the significant parameters. Blue color represents decrease in parameters value, while red represents increase in parameters value. This diagram shows the sensitivity of the ICER values upon changes in the model parameters. Value for each parameter is substituted one by one, starting with the lowest plausible value to the highest plausible value. Parameters that have the highest impact on the model are shown at the top, while the least impact is displayed at the bottom.

Figure 3 shows the results of Deterministic Sensitivity Analysis for TB screening among PL HIV against the prisoners as the reference strategy. Results showed that the ICER value never falls below zero after the iterations, indicating TB screening among PL HIV would remain relatively dominant in comparison to the reference strategy. The probability of TB detection among symptomatic PL HIV is shown to be the key driver for the ICER since it had the most impact on the ICER value. As the probability of TB detection among symptomatic PL HIV was increased to 0.0719 (+25%), the ICER value reduced to 389.67. Whereas, reduction of the same parameter to 0.0431 (−25%) would increase the ICER value to 6589.83. Results also showed that the cost for CXR did not affect the ICER value.

**Figure 4:**
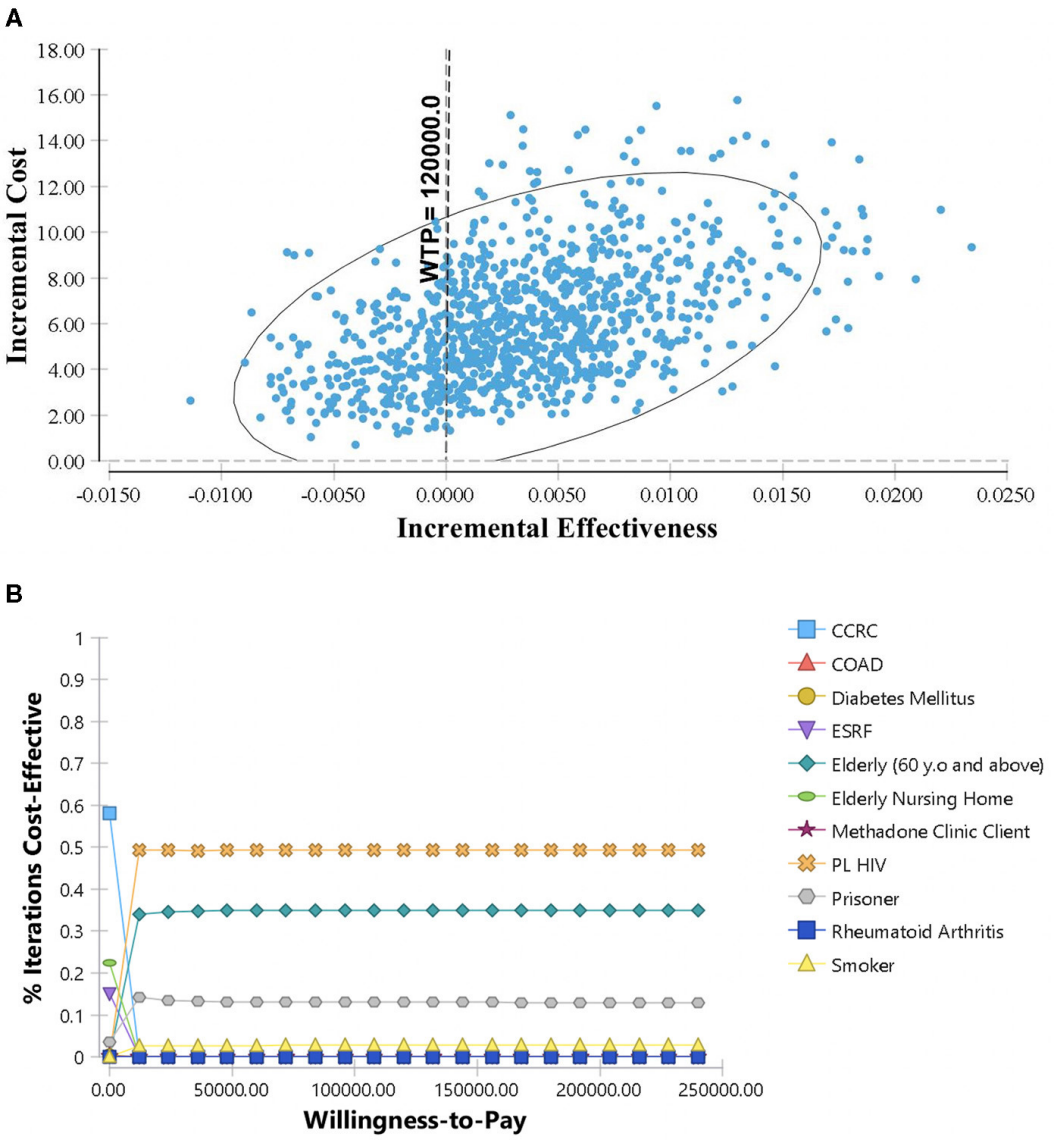
Probabilistic Sensitivity Analysis for TB screening among high-risk groups: **(A)** Scatter plot of incremental cost and incremental effectiveness for PL HIV vs. prisoners; **(B)** Cost-Effectiveness Acceptability Curve for high-risk groups TB screening. Each parameter in the model is assigned with suitable statistical distributions and allowed to diverse based on the corresponding distributions. The results of 1,000 simulations are shown in the cost-effectiveness plane as scatter plot of incremental cost and incremental effectiveness for PL HIV vs. prisoners. The Cost-Effectiveness Acceptability Curve (CEAC) shows the cost-effectiveness of screening among high-risk groups at various level of willingness to pay threshold.

PSA results for TB screening among the high-risk groups are demonstrated in Figure 4. The cost-effectiveness plane depicted 1,000 simulations of incremental cost and incremental effectiveness, which is the number of TB cases detected per 1,000 screenings. Almost 100% of the time, screening among PL HIV was more expensive compared to screening among prisoners. However, 74.3% of the iterations were in quadrant 1, which showed that screening among PL HIV was more effective than the prisoners. Whereas, the CEAC demonstrates the probability of screening among PL HIV was more effective compared to the prisoners throughout various willingness to pay threshold values until MYR 240,000. The results showed that screening among PL HIV was cost-effective around 48.6% of the iterations, almost at the full length of the corresponding values for WTP.

## Discussion

This study indicates that TB screening among PL HIV is the most cost-effective strategy. The result consistent with past studies, which revealed TB screening among HIV is cost-effective in both community and hospital settings (22, 23). HIV is a well-known risk factor for TB infection in low- and middle-income countries (1). In comparison to the non-HIV, there is a 16 to 27 times risk of getting TB infection among PL HIV. This is reflected in the prevalence of TB/HIV co-infection in Malaysia of 6% for 2018 (24). From the results of this study, it was also estimated that the cost to detect one TB case from PL HIV screening would be around MYR 2,597.00. The key driver for the cost-effectiveness model is the probability of TB cases detected among the symptomatic cases. The higher the probability of TB cases detected among the symptomatic, the lower the ICER; thus, the lower the cost for detecting one TB case.

In addition, TB screening among the elderly and prisoners also showed to be cost-effective. It would cost around MYR 2,868.62 and MYR 3,065.24 to detect one TB case by screening the elderly and prisoners, respectively. Studies were done in the US and Soviet Union also revealed similar results, in which screening of prisoners was more cost-effective than those of conventional community screening (2). The high prevalence of TB among the jailed population is well-documented in previous reports and studies (1). This is due to environmental conditions such as enclosed space and poor ventilation, which lead to poor air circulation and subsequently precipitate TB infection (25, 26). Apart from that, there was enough evidence to show that TB incidence increases with age. However, the TB problem among the elderly is likely underestimated due to the difficulty of diagnosing TB among the older age groups (27). Hence, there was a suggestion that TB screening among the elderly should focus on active case detection (28).

On the other hand, TB screening among Diabetic patient was shown to have the highest cost per one TB case detected among the high-risk groups, with MYR 13,214.26. This might be due to low TB case detection despite a large amount of screening done compared to the other high-risk groups. The association between TB and DM is well-documented. However, there are several well-known micro factors that precipitate TB infection in DM patients (29). For example, patients with uncontrolled glycaemic levels and low Body Mass Index (BMI) are known to have a higher risk of contracting TB (30). Thus, past studies recommend focusing on TB screening among low BMI, high Fasting Blood Sugar and low Triglycerides rather than the entire DM patients (31). Similarly, the cost per TB case detected was also high for CCRC inmates, with MYR 12,809.08. People Who Use Drugs (PWUD) are also known to be at higher risk for TB infection (32). Plus, living in a closed, packed and condensed environment such as in rehabilitation center put them at much higher risk for TB infection (25, 26). A study done on TB screening at substance abuse treatment centers in Malaysia revealed that the PWUD is at a much higher risk of Latent Tuberculosis Infection (LTBI), which can later progress into active disease (33). Nevertheless, the MOH report showed that only a small percentage was actually being diagnosed with TB (7).

The decision to focus TB screening on one strategy or to expand it to other strategies should depend on the ICER value. This study suggests that to implementation of TB screening among PL HIV will incur an additional cost per screening even though the benefit outweighs the reference strategy, i.e., TB screening among the prisoners. Hence, it would cost an additional MYR 735.82 to switch the strategy from prisoners to PL HIV with an additional one TB case being diagnosed. Considering the number of screening will affect the number of TB cases detected, the availability of those specific high-risk groups will affect how much it will cost for each TB screening strategy.

This study's main strength is the comprehensiveness of the analysis method with the inclusion of various high-risk groups. Hence, this study provides a better understanding of TB screening among the high-risk groups in terms of its' cost-effectiveness. While providing a better overview of each high-risk group's cost-effectiveness, this study will be useful for policy makers in strategizing future TB elimination program. Besides that, this study also received input from MOH and program owner, who directly involved in managing the TB screening program.

Notwithstanding the above, this study may provide significant input to the policy makers. Screening among high-risk groups has been recognized as the cornerstone for TB elimination (34). However, different strategies are required due to the variability in resource availability and disease transmission in local settings (35). In re-strategizing the national TB program, prioritization is necessary to make sure the current available resources are being allocated in the best possible manner. In a limited budget availability, focusing TB screening among the highly cost-effective strategies seems to be the way forward for the policymakers. For example, in Japan and the US, older people are given priority for TB screening (36). Hence, in Malaysia, TB screening among PL HIV, the elderly and prisoners should be the focus of the TB screening program as suggested by this study. In addition, this study is also useful for budgetary planning. By setting a target for TB case detection, the cost for each TB case detection can be used to estimate the required budget for TB program implementation. This study also adds to the current knowledge that TB screening strategies should differ from one country to another. Moreover, this study presents the costs of detecting a TB case for each high risk-groups in the context of Malaysia, which are not previously available for decision making. Hence, by utilizing the Cost-Effectiveness Analysis (CEA) model of multiple alternatives, this study provides a meaningful approach to prioritization of TB screening among high-risk groups, which might be useful to the academicians and also the health care professionals for on-field practices.

Nevertheless, there are several limitations to this study. The measure of effectiveness used in this study was generic, i.e., cost per TB case detected. Most of the cost-effectiveness studies among TB high-risk groups expressed the effectiveness measure using Quality Adjusted Life Years (QALY), Disability Adjusted Life Years (DALY) averted or death averted, but there are some which expressed the measure of effectiveness in term of TB cases detected (2). The use of cost per TB case detected as the measure of cost-effectiveness has a major limitation especially when applying the WTP threshold. Since there is no documented WTP threshold measured for TB cases detected, the WTP threshold used as a comparator may not reflect the real health opportunity cost. The conclusion also would likely change when using a more standardized outcome measure such as QALYs or DALYs. Since QALYs and DALYs value would probably be different between age groups, it would therefore change the outcome of this study especially for the elderly and those residing in the elderly nursing home (37). Besides, the lack of standardization for outcome measurement makes it difficult to compare the findings with other studies. The benefit of the current study might be overestimated or underestimated due to the use of these outcome measures. For example, the overestimation of benefit in screening among the elderly vs. younger age group due to the effect of time horizon analysis, as well as the screening for cases in confined space vs. non-confined space.

This study also did not take into account the potential TB cases averted among the high-risk group. For example, individuals living in institutions such as prisons or CCRC might contribute higher transmission compared to the PL HIV, elderly and others. Data used in this study also confined to Sabah and Sarawak states. Thus, probabilities for certain high-risk groups might not represent the exact probabilities for the country. This was particularly noticeable especially on probabilities for Elderly Nursing home residents, clients of Methadone Clinic, and Rheumatoid Arthritis patients. By using secondary data, the current study also limits further detailed analysis.

In conclusion, this study recommends prioritization on several high-risk groups in TB screening program based on the most cost-effective strategies, such as among PL HIV, elderly and prisoners. TB screening among other high-risk groups should be implemented based on the available resources. Therefore, to exercise a strategic plan for TB screening, the policy makers must also take into account the effect of these factors and how they will benefit in long run. It is suggested that detailed analysis be conducted in future studies, for example by looking at the cost-effectiveness of TB screening between different sub-groups of DM. Future research should also focus on screening for latent TB in Malaysia. Despite that, the current study suggests that re-strategizing TB screening program among high-risk groups should be the way forward. With the scarce resources and new modalities coming in for TB diagnosis, there is a need for prioritizing the TB screening program. Hence, the limited resources can be used for the most cost-effective measures and to tackle other issues, while moving forward into eliminating TB.

## Data Availability Statement

The original contributions presented in the study are included in the article/supplementary material, further inquiries can be directed to the corresponding author/s.

## Ethics Statement

The study was conducted according to the guidelines of the Declaration of Helsinki, and did not include any identifiable human data. The study had obtained approval from the Institutional Review Board (or Ethics Committee) of Ministry of Health Malaysia (NMRR-19-3443-51729).

## Author Contributions

NM: conceptualization, software, formal analysis, resources, writing—original draft preparation, visualization, project administration, and funding acquisition. NM, AR, and MS: methodology and investigation. MM, JH, NZ, MB, and FA: validation and writing—review and editing. NM, MS, and MM: data curation. All authors have read and agreed to the published version of the manuscript.

## Conflict of Interest

The authors declare that the research was conducted in the absence of any commercial or financial relationships that could be construed as a potential conflict of interest.

## References

[1] WHO. Global Tuberculosis Report 2019. Geneva: World Health Organization (2019). Available online at: https://apps.who.int/iris/bitstream/handle/10665/329368/9789241565714-eng.pdf (accessed January 1, 2020).

[2] Dobler CC. Screening strategies for active tuberculosis: focus on cost-effectiveness. Clin Econ Outcomes Res. (2016) 8:335–47. 10.2147/CEOR.S92244 27418848 PMC4934456

[3] Jiménez-Fuentes MA Milaauge C Gómez MNA Peiro JS de Souza Galvao ML Maldonado J. Screening for active tuberculosis in high-risk groups. Int J Tuberc Lung Dis. (2014) 18:1459–65. 10.5588/ijtld.14.0271 25517812

[4] Zenner D Southern J van Hest R DeVries G Stagg HR Antoine D. Active case finding for tuberculosis among high-risk groups in low-incidence countries. Int J Tuberc Lung Dis. (2013) 17:573–82. 10.5588/ijtld.12.0920 23575321

[5] Thammavong C Paboriboune P Bouchard B Harimanana A Babin F-X Phimmasone P. Bleach treatment of sputum samples aids pulmonary tuberculosis screening among HIV-infected patients in Laos. Int J Tuberc Lung Dis. (2011) 15:75. 10.5588/ijtld.11.0075 22283894

[6] de Siqueira-Filha NT Legood R Cavalcanti A Santos AC. Cost of tuberculosis diagnosis and treatment in patients with HIV: a systematic literature review. Value Health. (2018) 21:482–90. 10.1016/j.jval.2017.09.003 29680106

[7] MOH. Malaysia National Strategic Plan for Tuberculosis Control (2016–2020). Putajaya: MOH (2016). Available online at: http://www.moh.gov.mywebsites (accessed February1, 2020).

[8] MOH. urat Pekeliling KPK Bil 1 2016 - Pengukuhan Saringan Golongan Berisoko Tinggi Tuberculosis (Tibi) di bawah Program Kawalan Tibi Kebangsaan KKM -.pdf. Putajaya: MOH (2016).

[9] Wingate LT Coleman MS Posey DL Zhou W Olson CK Maskery B. Cost-effectiveness of screening and treating foreign-born students for tuberculosis before entering the United States. PLoS ONE. (2015) 10:e0124116. 10.1371/journal.pone.0124116 25924009 PMC4414530

[10] Andrews JR Lawn SD Rusu C Wood R Noubary F Bender MA. The cost-effectiveness of routine tuberculosis screening with Xpert MTB/RIF prior to initiation of antiretroviral therapy. AIDS. (2012) 26:987–95. 10.1097/QAD.0b013e3283522d47 22333751 PMC3517815

[11] Nishikiori N van Weezenbeek C. Target prioritization and strategy selection for active case-finding of pulmonary tuberculosis: a tool to support country-level project planning. BMC Public Health. (2013) 13:97. 10.1186/1471-2458-13-97 23374118 PMC3602078

[12] Ahmad Hanis AS Zahiruddin WM Zahariyah Y Khairolnizam I Malek SN Maimunah S. Associated factors for positive CXR among TB high risk group screening in kedah unmet health need among elderly with diabetes in Penang: a mixed method study view project. J Biomed Clin Sci. (2019) 4:11–5.

[13] Smit GSA Apers L de Onate WA Beutels P Dorny P Forier AM. Cost-effectiveness of screening for active cases of tuberculosis in flanders, Belgium. Bull World Health Org. (2017) 95:27–35. 10.2471/BLT.16.169383 28053362 PMC5180339

[14] Kranzer K Afnan-Holmes H Tomlin K Golub JE Shapiro AE Schaap A. The benefits to communities and individuals of screening for active tuberculosis disease: a systematic review. Int J Tuberc Lung Dis. (2013) 17:432–46. 10.5588/ijtld.12.0743 23485377

[15] Bank Negara Malaysia (BNM). Exchange Rates. (2021). Available online at: https://www.bnm.gov.my/exchange-rates (accessed Junuary 1, 2021).

[16] WHO Commission on Macroeconomics and Health & World Health Organization. Macroeconomics and Health: Investing in Health for Economic Development: Executive Summary/Report of the Commission on Macroeconomics and Health. World Health Organization (2001). Available online at: https://apps.who.int/iris/handle/10665/42463 (accessed February 1, 2020).

[17] Marseille E Larson B Kazi DS Kahn JG Rosen S. Thresholds for the cost-effectiveness of interventions: alternative approaches. Bull World Health Org. (2015) 93:118–24. 10.2471/BLT.14.138206 25883405 PMC4339959

[18] International Monetary Fund. GDP per Capita, Current Prices. World Economic Outlook (2018). Available online at: https://www.imf.org/external/datamapper/PPPPC@WEO/THA (accessed February1, 2020).

[19] MOH. Management of Tuberculosis. 3rd ed. Putrajaya: MOH (2012).

[20] Baio G Dawid AP. Probabilistic sensitivity analysis in health economics. Stat Methods Med Res. (2015) 24:615–34. 10.1177/0962280211419832 21930515

[21] Andronis L Barton P Bryan S. Sensitivity analysis in economic evaluation: an audit of NICE current practice and a review of its use and value in decision-making. Health Technol Assess. (2009) 13:iii, ix–xi, 1–61. 10.3310/hta13290 19500484

[22] Zwerling A. Costs of tuberculosis screening among inpatients with HIV. Lancet Global Health. (2019) 7:e163-4. 10.1016/S2214-109X(18)30564-3 30683225

[23] Gilbert JA Shenoi SV Moll AP Friedland GH Paltiel AD Galvani AP. Cost-effectiveness of community-based TB/HIV screening and linkage to care in rural South Africa. PLoS ONE. (2016) 11:e0165614. 10.1371/journal.pone.0165614 27906986 PMC5131994

[24] Ministry of Health. Country Progress Report on HIV/AIDS (2018). p. 1–45. Available online at: http://www.moh.gov.my/resources/index/Penerbitan/Laporan/Report_GAM_2018_Final_(2).pdf (accessed February1, 2020).

[25] Migliori GB Ambrosio LD Centis R van den Boom M Ehsani S Dara M. Guiding Principles to Reduce Tuberculosis Transmission in the WHO European Region. WHO (2018). p. 1–51. Available online at: http://www.euro.who.int/pubrequest (accessed February 1, 2020).

[26] CDC. CDC Infectious Control. p. 189–226. Available online at: https://www.cdc.gov/tb/education/corecurr/pdf/chapter7.pdf (accessed February 1, 2020).

[27] Marchand R Tousignant P Chang H. Cost-effectiveness of screening compared to case-finding approaches to tuberculosis in long term care facilities for the elderly. Int J Epidemiol. (1999) 28:563–70. 10.1093/ije/28.3.563 10405865

[28] Lee SH. Active case finding in the elderly tuberculosis in South Korea. Tuberc Resp Dis. (2019) 82:261. 10.4046/trd.2019.0043 31267723 PMC6609521

[29] Lee PH Fu H Lee MR Magee M Lin HH. Tuberculosis and diabetes in low and moderate tuberculosis incidence countries. Int J Tuberc Lung Dis. (2018) 22:7–16. 10.5588/ijtld.17.0329 29297421

[30] Lee PH Fu H Lai TC Chiang CY Chan CC Lin HH. Glycemic control and the risk of tuberculosis: a cohort study. PLoS Med. (2016) 13:e1002072. 10.1371/journal.pmed.1002072 27505150 PMC4978445

[31] Ji Y Cao H Liu Q Li Z Song H Xu D. Screening for pulmonary tuberculosis in high-risk groups of diabetic patients. Int J Infect Dis. (2020) 93:84–9. 10.1016/j.ijid.2020.01.019 31978585

[32] Nava-Aguilera E Andersson N Harris E Mitchell S Hamel C Shea B. Risk factors associated with recent transmission of tuberculosis: systematic review and meta-analysis. Int J Tuberc Lung Dis. (2009) 13:17–26. 19105874

[33] Al-Darraji HAA Wong KC Yeow DGE Fu JJ Loeliger K Paiji C. Tuberculosis screening in a novel substance abuse treatment center in Malaysia: implications for a comprehensive approach for integrated care. J Subs Abuse Treat. (2014) 46:144–9. 10.1016/j.jsat.2013.08.023 24074846 PMC5189907

[34] Jamison DT Breman JG Measham AR Alleyne G Claeson M Evans DB. Disease Control Priorities in Developing Countries. 2nd ed. Oxford: Oxford University Press (2006). 21250309

[35] Menzies NA Gomez GB Bozzani F Chatterjee S Foster N Baena IG. Cost-effectiveness and resource implications of aggressive action on tuberculosis in China, India, and South Africa: a combined analysis of nine models. Lancet Global Health. (2016) 4:e816–26. 10.1016/S2214-109X(16)30265-0 27720689 PMC5527122

[36] Chong KC Leung CC Yew WW Zee BCY Tam GCH Wang MH. Mathematical modelling of the impact of treating latent tuberculosis infection in the elderly in a city with intermediate tuberculosis burden. Sci Rep. (2019) 9:4869. 10.1038/s41598-019-41256-4 30890762 PMC6424958

[37] Saxena N Sethia D. Decomposition of years of life lost due to premature death (YLL): a method for spatial and temporal comparative assessment. Arch Public Health. (2020) 78:91. 10.1186/s13690-020-00472-5 33042537 PMC7541272

